# Heat Waves and Climate Change: Applying the Health Belief Model to Identify Predictors of Risk Perception and Adaptive Behaviours in Adelaide, Australia

**DOI:** 10.3390/ijerph10062164

**Published:** 2013-05-29

**Authors:** Derick A. Akompab, Peng Bi, Susan Williams, Janet Grant, Iain A. Walker, Martha Augoustinos

**Affiliations:** 1 Discipline of Public Health, School of Population Health, The University of Adelaide, Adelaide, SA 5005, Australia; E-Mails: derick.akompab@adelaide.edu.au (D.A.A); susan.williams@adelaide.edu.au (S.W.); 2 Population Research & Outcome Studies, The University of Adelaide, Adelaide, SA 5005, Australia; E-Mail: janet.grant@adelaide.edu.au; 3 Climate Adaptation Flagship, Commonwealth Scientific and Industrial Research Organisation, CSIRO, Perth, WA 6931, Australia; E-Mail: iain.a.walker@csiro.au; 4 School of Psychology, The University of Adelaide, Adelaide, SA 5005, Australia; E-Mail: martha.augoustinos@adelaide.edu.au

**Keywords:** climate change, heat waves, health belief model, risk perception, adaptive behaviours, Australia

## Abstract

Heat waves are considered a health risk and they are likely to increase in frequency, intensity and duration as a consequence of climate change. The effects of heat waves on human health could be reduced if individuals recognise the risks and adopt healthy behaviours during a heat wave. The purpose of this study was to determine the predictors of risk perception using a heat wave scenario and identify the constructs of the health belief model that could predict adaptive behaviours during a heat wave. A cross-sectional study was conducted during the summer of 2012 among a sample of persons aged between 30 to 69 years in Adelaide. Participants’ perceptions were assessed using the health belief model as a conceptual frame. Their knowledge about heat waves and adaptive behaviours during heat waves was also assessed. Logistic regression analyses were performed to determine the predictors of risk perception to a heat wave scenario and adaptive behaviours during a heat wave. Of the 267 participants, about half (50.9%) had a high risk perception to heat waves while 82.8% had good adaptive behaviours during a heat wave. Multivariate models found that age was a significant predictor of risk perception. In addition, participants who were married (OR = 0.21; 95% CI, 0.07–0.62), who earned a gross annual household income of ≥$60,000 (OR = 0.41; 95% CI, 0.17–0.94) and without a fan (OR = 0.29; 95% CI, 0.11–0.79) were less likely to have a high risk perception to heat waves. Those who were living with others (OR = 2.87; 95% CI, 1.19–6.90) were more likely to have a high risk perception to heat waves. On the other hand, participants with a high perceived benefit (OR = 2.14; 95% CI, 1.00–4.58), a high “cues to action” (OR = 3.71; 95% CI, 1.63–8.43), who had additional training or education after high school (OR = 2.65; 95% CI, 1.25–5.58) and who earned a gross annual household income of ≥$60,000 (OR = 2.66; 95% CI, 1.07–6.56) were more likely to have good adaptive behaviours during a heat wave. The health belief model could be useful to guide the design and implementation of interventions to promote adaptive behaviours during heat waves.

## 1. Introduction

Climate change has been projected to increase the frequency and severity of extreme weather events such as heat waves [1,2]. Heat waves, characterised by stagnant warm air masses and consecutive nights with high temperatures [3] are considered a public health problem since they are associated with heat-related morbidity and mortality [4]. In 2003, Western Europe experienced one of its worst heat waves which resulted in an estimated 70,000 heat-related deaths [5]. France was the worst affected country with over 14,800 recorded heat-related deaths [6,7]. Heat waves are the leading cause of weather-related deaths in the United States with an estimated 688 deaths reported to be directly related to heat each year [8]. In 2010, Russia experienced an unprecedented heat wave that resulted in approximately 15,000 heat-related deaths [9].

In Australia, a number of cities usually experience heat waves during summer, which are considered to have claimed more lives than any other natural disaster in the country [10]. It is estimated that over 1,000 heat-related deaths occur in Australia every year [11]. In the summer of 2009, south-eastern Australia experienced an extreme heat wave between 27 January and 8 February which resulted in an estimated 500 heat-related deaths in Adelaide and Melbourne [12]. During the early 2009 heat wave, there was an increase in direct heat-related hospital admissions, emergency department presentations for ischaemic heart disease and increased ambulance call-outs for cardiac-related diseases in Adelaide [13]. Most recently, between late 2012 and early 2013, Australia was gripped with an extreme heat wave that caused temperature rises across large parts of Western Australia, Queensland, Victoria, South Australia, New South Wales, the Northern Territory and Tasmania; daily maximum temperatures of over 40 °C were recorded in some of the cities across the country. The extreme temperatures resulted in widespread bushfires and the loss of livelihoods [14,15,16]. In South Australia, the Bureau of Meteorology defines a heat wave as a period of maximum temperatures of 35 °C or over for a period of five or more consecutive days or three or more consecutive days of temperatures of 40 °C or above [17]. 

There has been increasing concern among authorities on the dangers of heat waves to public health and safety. However, there is still a lack of recognition among the public about the social and health consequences associated with heat waves [18]. There are a number of factors that can increase human vulnerability to heat-related morbidity and mortality [19,20,21,22]. However, heat-related illnesses and deaths can be largely prevented if individuals recognise the risks and undertake simple behaviour modification measures such as seeking cool shelters, reducing physical activity, drinking sufficient water and staying in an air-conditioned environment [23,24]. 

Heat waves are an environmental hazard and there are a number of socio-demographic factors that may influence risk perception to natural hazards [25,26,27]. For example, age has been found to be positively correlated with risk perception to hazards [28], although negative correlations have also been reported [29]. Gender is often found to be an important determinant of risk perception as women perceive risks more than men [30]. In addition, individuals with a high income and education level have been found to have a lower perception of risks [31,32]. In relation to heat waves, the extent to which an individual perceives the risks associated with heat waves may determine how he/she takes action to prevent heat-related morbidity or mortality during heat waves. A study conducted in North America to gauge public perception of heat waves revealed that a majority of participants did not consider themselves as vulnerable [18]. Similar findings were obtained in the United Kingdom [33], where the elderly who are known to be vulnerable to heat waves did not perceive themselves as being at risk during a heat wave. This has implications for risk communication strategies during heat waves and could limit the effectiveness of current heat-health warning systems and advisories. It highlights the importance of understanding the social and cognitive factors that determine risk perception and behavioural adaptation during heat waves. 

There is currently a growing interest in using socio-cognitive theories to study risk perception and motivation for adaptation to climate-related risks since knowledge and perceptions can drive behaviour change [34]. Despite the health risks that heat waves pose for Australians, there has been very limited research examining the social and cognitive factors that influence risk perception and behavioural response during a heat wave. There are a number of behaviour change models that could be used to examine how knowledge and perception can influence behaviour modification in relation to health risks. For example, the health belief model (HBM) could be applied as the theoretical framework to examine how cognitive factors shape an individual’s perception and hence adaptive behaviours during heat waves. This model focuses on the beliefs of individuals to explain health behaviours [35] and has been used to guide the development of behaviour change programs [36]. The model is based on a value expectancy approach [37] and postulates that human behaviour depends mainly on the value that an individual places on a particular outcome and on the individual’s estimate of the likelihood that a given action will achieve that outcome [38]. The model suggests that whether or not individuals take action to protect their health is a function of whether they believe that (a) they are susceptible to a particular threat (b) that such a threat would have severe consequences, (c) that they have options available to them to take preventive action, and (d) that the benefits of taking preventive action outweigh the costs [39]. The main constructs of the HBM used in this study are perceived susceptibility, perceived severity, perceived benefit of taking preventive measures, perceived barriers to the preventive action and cues to taking action (the presence of information and other triggers that motivates undertaking preventive action). A combination of perceived susceptibility and perceived severity has been termed “perceived threat” [40], which in this paper is known as risk perception. In this paper, we have used the phrase “perceived vulnerability” in place of “perceived susceptibility”, since vulnerability is widely used in the literature on heat waves. Furthermore, the HBM was selected for this study because some of its constructs relate to perception, part of the focus of the study.

The objectives of this study were to: (a) to examine the usefulness of the constructs of the HBM in predicting the adoption of healthy behaviours during heat waves, (b) to identify the factors that will predict risk perception to heat waves, (c) to assess participants’ knowledge related to heat waves. We hypothesised that if participants perceive heat waves as a health risk, then the constructs of the HBM might be able to predict adaptive behaviours during periods of heat waves. These findings could be important in improving risk communication and promoting behaviour change strategies during heat waves. This paper reports part of the findings of a larger study conducted in Adelaide. The results of the other part have been reported elsewhere [41].

## 2. Materials and Methods

### 2.1. Study Setting and Data Collection

This cross-sectional study was conducted in the city of Adelaide, the capital of South Australia. South Australia has an estimated population of 1.64 million inhabitants with over three-quarters of the state’s population residing in Adelaide [42]. The city usually experiences hot dry summers, with the months of January to March being the hottest in the year and heat waves are quite common. Recently in early 2013, Adelaide experienced hot spells over a couple of days with a daily maximum temperature of 45 °C recorded on January 4th, 2013 [43]. However, the most recent severe heat waves occurred in 2008, 2009 and 2010 [13,17,44]. There were two heat waves recorded in 2009; the first between late January and early February and the second in November [17]. During the early 2009 heat wave, there were 13 consecutive days with temperatures above normal (monthly average temperatures normally range from approximately 27 °C to 29.4 °C during summer); six of these days had temperatures above 35 °C and a record temperature of 45.7 °C was recorded in one of the days [13,45].

The approach by which the participants were recruited and how data were collected has previously been described [41]. Briefly, participants for the study were recruited from the North West Adelaide Health Study (NWAHS) cohort, which is a random representative sample of residents in the north and western parts of Adelaide [46]. With support of the chief investigators of this cohort, and as part of a follow-up survey administered by the NWAHS in late 2011 among a sub-group of the cohort, study participants were asked if they would be willing to be contacted at a later date to take part in a questionnaire study on heat waves. The age profile of the sub-group approached did not include older participants. It should be noted that the representation of the elderly in the NWAHS cohort is decreasing, because of continual movement of this group to assisted care facilities due to health reasons. Of the 1,185 participants approached, 818 expressed interest to participate in the heat waves study. Among those who expressed interest, 490 participants were selected after excluding those with insufficient literacy skills or long term illness.

A package containing an approach letter, the study information sheet, the self-administered questionnaire and a reply paid envelope was mailed to the 490 individuals who were selected to take part in the study. Questionnaires were mailed out to selected participants during the third week of January 2012, following three weeks of hot weather in Adelaide. The timing was considered optimal because we wanted participants’ responses to reflect their experiences of the hot weather. For example, the daily maximum temperature recorded in Adelaide on 1 January 2012 was 41.6 °C, making it the warmest start to the year for over a century [47]. The latter part of the month of January remained hot with eleven consecutive days of temperatures over 30 °C recorded between the 19th to the 29th including four consecutive days with temperatures above 35 °C from the 22nd to the 25th [48]. There were no follow-up or reminder calls made to participants. Ethics clearance was obtained from The University of Adelaide Human Research Ethics Committee (No. H-061-2011) and The Queen Elizabeth Hospital Human Research Committee (No. 2011136). Ethics clearance was obtained from the latter institution because it over sees any research study involving the cohort. 

### 2.2. Measures and Classification

The questionnaire collected data on participants’ demographic and household characteristics, knowledge about heat waves, perceptions of a heat wave and adaptive behaviours during a heat wave. Perceptions were measured in the context of a heat wave scenario using the HBM as the theoretical framework. Although it would have been ideal to conduct the study during a heat wave, the logistics of implementing the study to coincide with a heat wave was considered to be too difficult, so a hypothetical scenario was formulated.

***Demographic and household characteristics:*** The questionnaire collected data on the following variables: age, gender, marital status, educational level, employment status, gross annual household income, living arrangements, fan and air-conditioner ownership.

***Knowledge about heat waves:*** Knowledge about heat waves (Cronbach’s alpha = 0.69) was measured with eight statements; four of which were negatively worded. The response choices were “True”, “False” or “Don’t know”. For the positively worded statements, all the “True” responses were given a score of “1” while the “False” and “Don’t know” responses were given a score of “0”. For the negatively worded statements, all the “False” responses were given a score of “1” and the “True” and “Don’t Know” responses were given a score of “0”. The knowledge scores were then summed to obtain a “total knowledge score” with a maximum possible score of eight. We dichotomised knowledge about heat waves into low (1–4) and high (5–8) at approximately the “midpoint” of the total score as shown in Table 1.

***Perception of a heat wave scenario (as applied in the HBM):*** A heat wave scenario with 45 °C for seven consecutive days was used to measure participants’ risk perception. We used an extreme heat scenario since we wanted to evaluate how participants conceptualise the risk posed by a health threat. Nonetheless, the scenario could be considered plausible in comparison to the heat wave experienced in early 2009. 

We provided participants with a number of statements about what might happen to them under such a heat wave scenario and they were requested to provide responses on a 5-point Likert scale (*i.e.*, Strongly Disagree, Disagree, Unknown, Agree, Strongly Agree; scored from “1” to “5” respectively). There were a number of statements for each of the constructs of the HBM (perceived vulnerability, perceived severity, perceived threat, perceived benefit, perceived barrier and cues to action). We then added participants’ total score for each of the constructs and later dichotomised (low/high) at approximately the “mid-point” of the total score (see Table 1).

**Table 1:** Range and classification of scores for heat wave knowledge, perceptions and adaptive behaviours.

Key variables	Range of total scores	Classification	
		LOW	HIGH
Knowledge about heat waves	1–8	1–4	5–8
Perceived vulnerability	5–20	5–12	13–20
Perceived severity	5–25	5–15	16–25
Risk perception ^1^	70–500	70–251	252–500
Perceived benefit	15–30	15–22	23–30
Perceived barriers	4–16	4–10	11–16
Cues to action	8–25	8–16	17–25
Adaptive behaviours ^2^	15–29	15–21	22–29

^1^ Risk perception was computed by multiplying each participant’s total score for perceived vulnerability and perceived severity. As shown in the table, the minimum score for risk perception was 70 because this participant had a total score of 5 and 14 for perceived vulnerability and perceived severity respectively. No participant had a total score of 5 for perceived vulnerability and perceived severity respectively, so the lowest value cannot be 25; ^2^ Also referred to as poor (low) and good (high) adaptive behaviours. Those with poor adaptive behaviours may be considered to be at risk since they have poor preventive behaviours during a heat wave; behaviour change programs should therefore target such group of individuals.

Perceived vulnerability (Cronbach’s alpha = 0.75) to the effects of the heat wave scenario was measured by summing participants’ responses on four statements e.g., “*I think I may suffer from dehydration during such a heatwave period*”. Perceived severity (Cronbach’s alpha = 0.74) of the effects of the heat wave was measured by summing participants’ responses on five statements e.g., “*If I get dehydrated during such a heat wave period, it may lead me to being hospitalised*”. We calculated risk perception by multiplying each participant’s total scores for perceived vulnerability and perceived severity. We then dichotomised the total score for risk perception (low/high) using the median split to generate a dichotomous variable (see Table 1). Perceived benefit (Cronbach’s alpha = 0.87) was measured by summing participants’ responses on six statements, three of which were negatively worded. The scores of the negatively worded statements were reversed before the total scores were summed, e.g., “*Staying in an air conditioned environment will reduce the chance of me suffering from dehydration*”. Perceived barriers (Cronbach’s alpha = 0.73) to taking preventive action to the health effects of the heat wave was measured by summing participants’ responses on four statements, e.g., “*For security reasons, I would not open my windows at night to allow air to enter during such a heatwave*”. Cues to action (Cronbach’s alpha = 0.83) was measured by summing participants’ responses on five statements, e.g., “*I will take precautionary measures if my doctor advises me about the dangers of the heat wave*”. 

***Adaptive behaviours during a heat wave****:* Participants were asked how often they undertook a number of behavioural adaptations during a heat wave. There were ten statements to assess adaptive behaviours (Cronbach’s alpha = 0.60), three of which were negatively worded: “*Wear-dark coloured clothes when going outside*”, “*Drink a few cups of coffee to stay alert*”, “*Do some outdoor gardening during the day*”. The statements related to adaptive behaviours had the scores and response choices (1 = Never, 2 = Sometimes, 3 = Always). We reversed the scores of the negatively worded statements and then added the total score for adaptive behaviours. We then dichotomised the total adaptive behaviours score into two levels at approximately the “mid-point”: good (22–29) and poor (15–21) (see Table 1), generating a dichotomous variable for adaptive behaviours during a heat wave.

## 3. Data Analysis

Of the 490 questionnaires mailed out to participants, 272 were returned, giving a response rate of 55.5%. The questionnaires were checked for accuracy, consistency and completeness. Five were discarded due to missing data, leaving 267 for final analysis. Respondents and non-respondents differed only by age. Data were analysed with Stata version 12 (Stata Corp., College Station, TX, USA) and descriptive statistical techniques were used to determine frequency distributions while logistic regression was used to identify significant relationships among variables. 

In order to identify the predictors of risk perception (low, high) to heat waves, the predictor variables were age as a continuous variable, gender, marital status, educational level, employment status, gross annual household income, living arrangements, fan ownership, air-conditioner ownership and knowledge about heat waves as a continuous variable. Risk perception was coded as a binary variable with “low” coded as “0” and “high” coded as “1”. First, univariate logistic regression analyses were performed for all the predictor variables. Next, multivariate logistic regression analysis was performed to identify the independent predictors of risk perception to heat waves, controlling for confounding effects.

To examine the usefulness of the constructs of the HBM in predicting adaptive behaviours during heat waves, the dependent variable was adaptive behaviours in the dichotomous form (poor, good), while the predictor variables were risk perception, perceived benefit, perceived barrier, cues to action, with knowledge about heat waves as a continuous variable, demographic and household characteristics as covariates. Adaptive behaviours was coded as a binary variable with “poor” coded as “0” and “good” coded as ‘1”. Initially, univariate logistic regression analyses were performed for all the potential predictor variables of adaptive behaviours. Next, a multivariate logistic regression model was performed that consisted of risk perception, perceived benefit, perceived barrier, cues to action and the demographic variables that were significant predictors in univariate analyses. We included only the significant demographic variables from univariate analyses into the multivariate model in order to avoid over fitting the model due to the limited data points in the lower category of adaptive behaviours. Due to sample size constraints some categorical demographic variables (e.g., marital status, level of education, employment status) were dichotomised. We used a two-sided *p* < 0.05 to determine statistical significance in all analyses. 

## 4. Results

### 4.1. Demographic and Household Characteristics of Study Participants

Table 2 shows the demographic and household characteristics of the study participants. The mean age of the participants was 51 years (SD, 8.7). When collapsed into two groups, 61.4% belong to the older age group (50–69 years) while the remainders (38.6%) belong to the younger age group (30–49 years). More than half (55.8%) of the participants were females. 

**Table 2:** Demographic and household characteristics of the study participants.

Variable		Number	Percent(% )
Age group (n = 267) (Mean = 51 years)			
	30–49	103	38.6
	50–69	164	61.4
Gender (n = 267)			
	Male	118	44.2
	Female	149	55.8
Marital status (n = 267)			
	Never married	29	10.9
	Married ^1^	238	89.1
Level of education (n = 267)			
	Partial or full completion of high school	103	38.6
	Additional training after high school ^2^	164	61.4
Employment status (n = 267)			
	Not employed ^3^	43	16.1
	Employed (full/part time, self employed)	224	83.9
Gross annual household income (n = 254)			
	<$40,000	58	22.8
	$40,000–$59,999	53	20.9
	≥$60,000	143	56.3
Living arrangements (n = 267)			
	Alone	44	16.5
	With others^ 4^	223	83.5
Fan ownership (n = 265)			
	Yes	239	90.2
	No	26	9.8
Air conditioner ownership (n = 267)			
	Yes	250	93.6
	No	17	6.4

N/B: This table is a modified and extended version of that originally published in a separate paper. See reference [41]. ^1^ Married referred to those who have been married at some stage in life and include those married at the time of the study, separated or widowed. ^2^ Training after high school, bachelor or postgraduate degree. ^3^ Not employed referred to unemployed, retired, home duties or other. ^4^ Living with others referred to living with partner, family, relatives or other people.

A majority (89.1%) were married while 10.9% had never married. In terms of level of education, 38.6% had completed or partially completed high school while 61.4% had additional training after high school (such as apprenticeship, a trade certificate, a bachelor or postgraduate degree). Most of the participants (83.9%) were employed while 16.1% were either unemployed or retired. With regard to self-reported gross annual household income, 56.3% earned ≥$60,000, 20.9% earned between $40,000 and $59,999, and 22.8% earned less than $40,000. Most of the participants (83.5%) were living with others (*i.e.*, with either their partner, family or relatives); 16.5% were living alone. The majority of participants (90.2%) reported owning a fan, while 93.6% had an air-conditioner.

### 4.2. Knowledge about Heat Waves

A majority of the participants (91.4%) had good knowledge about heat waves. In terms of individual statements used to quantify knowledge, over half (51.7%) indicated that “*High atmospheric pressure with less rainfall could be responsible for extreme heat events in Adelaide*”. More than two-thirds indicated that “*Heat-related illnesses result from exposure to extreme heat*”. The detailed responses to the statements on knowledge about heat waves are shown in Table 3.

**Table 3:** Participants’ responses to the statements regarding knowledge about heat waves.

Statements	Number (% )		
	True	False	Don’t Know
High atmospheric pressure with less rainfall could be responsible for heat waves	138(51.7)	24(9.0)	105(39.3)
Heat-related illnesses results from extreme heat exposure	237(88.8)	20(7.5)	10(3.7)
Diabetes is an example of a heat-related illness	5 (1.9)	231(86.5)	31(11.6)
Excess sweating during a heat wave may be a sign of heat stress	154(57.7)	83(31.1)	30(11.2)
Individuals with heart conditions have a greater chance of becoming ill during a heat wave	196(73.4)	25(9.4)	46(17.2)
The elderly and young are the only ones vulnerable during a heat wave	23(8.6)	244(91.4)	0(0)
Heat-related illnesses are not known to cause deaths	29(10.9)	234(87.6)	4(1.5)
Heat waves may lead to bush fires	256(95.9)	8(3.0)	3(1.1)

### 4.3. Perception of a Heat Wave Scenario

Table 4 shows the overall classification of participants’ perceptions of a heat wave scenario (seven consecutive days with temperatures of 45 °C). Overall, 67.4% of the participants had a “high perceived vulnerability” under such a heat wave scenario, 76.4% of the participants were classified as having a “high perceived severity”, while 64.3% were classified as having a “high perceived benefit”. A majority of the participants (84.6%) were classified as having a “low perceived barrier” and 74.1% as having a “high cues to action”. In terms of risk perception, 50.9% of the participants had a high risk perception to the heat wave.

**Table 4:** Classification of participants’ perceptions in the context of a heat wave scenario based on the HBM and using the cut-off points.

Level of perceptions	Low		High	
	n	%	n	%
Perceived vulnerability	87	32.6	180	67.4
Perceived severity	63	23.6	204	76.4
Risk perception	131	49.1	136	50.9
Perceived benefit	95	35.7	171	64.3
Perceived barriers	226	84.6	41	15.4
Cues to action	69	25.9	197	74.1

**Table 5:** Participants’ responses to statements on perceptions in the context of a heat wave scenario using constructs of the health belief model.

Statements on perceptions (Five constructs of the HBM)		Percent * (%)				
		SA	A	U	D	SD
Perceived vulnerability	I think I may suffer from dehydration during such a heat wave	13.1	31.1	15.4	26.6	13.9
	I think my body temperature may rise abnormally during such a heat wave	13.9	38.2	19.5	21.3	7.1
	I think I may suffer from body weakness during such a heat wave	12.7	48.4	20.6	15.7	2.6
	I think I may suffer from sun burn if I get exposed during such a heat wave	43.6	40.8	5.9	6.7	3.0
Perceived severity	If my body temperature gets elevated during such a heat wave it may cause me to see a doctor	5.9	30.7	29.6	28.5	5.25
	If I get dehydrated during such a heat waves, it may lead me to being hospitalised	12.4	43.1	16.9	21.4	6.4
	Dehydration under such a heat wave may provoke long term damage to my health	13.1	42.7	24.7	16.9	2.6
	If I develop sun burn during such a heat wave, it may lead to skin cancer	29.2	52.8	12.4	4.1	1.5
	Hospitalisation as a result of dehydration during such heat wave may cause me to be absent from work	23.6	60.3	8.9	4.9	2.3
Perceived benefit	Eating hot meals during such a heat wave will enable me to cope with the heat	0.0	6.0	25.8	45.3	22.9
	Staying in an air conditioned environment will reduce the chance of me suffering from dehydration	31.5	47.9	8.2	10.1	2.3
	Using sunscreen will prevent me from developing sunburn during such a heat wave	25.1	51.7	8.6	13.1	1.5
	Wearing dark clothing outside during this period will reduce my chances of sweating	0.8	2.3	13.1	45.7	38.2
	Listening to daily weather forecasts would enable me to plan my outdoor activities	42.3	53.2	2.3	1.1	1.1
	Staying indoors during such a heat wave would be quite boring	8.7	29.3	19.6	34.6	7.7
Perceived barriers	Taking a cool shower from time to time at home during this period would waste water and increase my water bills	3.0	17.9	25.5	41.2	12.4
	For security reasons, I would not open my doors at night to allow air to enter during such a heat wave	5.6	15.7	10.1	49.8	18.7
	Due to my health, I will drink less water during such a heat wave	1.5	1.1	1.9	29.2	66.3
	Because of the cost of electricity, I would be reluctant to turn on the air conditioner during such a heat wave	2.6	4.5	4.1	41.6	47.2
Cues to action	A family member or friend tells me about the dangers of the heat wave	15.7	48.3	23.2	10.1	2.6
	I watch TV and see how an ambulance transports someone to the hospital due to dehydration from the heat wave	11.7	40.9	24.8	19.2	3.9
	I read a local news paper and get news about the health effects of the heat wave	11.3	53.0	23.3	10.5	1.9
	My doctor reminds me about the dangers of the heat wave	14.7	48.1	23.7	11.3	2.3
	As a result of my personal experience of heat waves in Adelaide, I would keep safe during such a heat wave	44.2	50.3	3.8	1.1	0.4

SA = Strongly Agree (5), A = Agree (4), U = Uncertain (3), D = Disagree (2), SD = Strongly Disagree (1); * Some percentages may not perfectively add up to 100% due to approximation to one decimal place.

Table 5 shows the details of the participants’ responses to the statements on perceptions in the context of the heat wave scenario. For example, 31.1% agreed that they might suffer from dehydration during a heat wave with temperature of 45 °C for seven consecutive days. Furthermore, 38.2% agreed that their body temperature would rise abnormally during such a heat wave. Also, 52.8% agreed that if they suffer from sun burn during such a heat wave, it may lead to skin cancer. In terms of participants’ responses to statements on “Cues to action” *i.e.*, what would motivate them to protect themselves from the harmful effects of such an extreme heat wave, there was a high level of participant agreement for most of these statements. For example, 53.0% agreed that they would adopt healthy behaviours if they read on a local newspaper about the health effects of the heat wave. Furthermore, 50.3% agreed with the statement that: “*As a result of my personal experience of heat waves in Adelaide, I would keep safe during such a heat wave*”. 

### 4.4. Adaptive Behaviours during a Heat Wave

Table 6 shows the details of participants’ responses to the statements used to quantify their adaptive behaviours during a heat wave. The first three adaptive behaviours with the highest percentage that were reported as “Always” performed were: drinking plenty of water to stay hydrated (83.8%), seeking protection of shady areas when outdoors (82.0%) and listening to daily weather forecasts (61.4%). The adaptive behaviour with the highest percentage that was reported as “Never” performed was: doing some outdoor gardening during the day (76.8%). A total adaptive behaviours score was calculated for each participant and their overall adaptive behaviour was classified as good (22–29) or poor (15–21) as shown in Table 1. Overall, 82.8% of the participants had “good adaptive behaviours” while 17.2% had “poor adaptive behaviours”.

**Table 6:** Participants’ responses to statements on adaptive behaviours.

Statement(s)	Percent (%)		
	Always	Sometimes	Never
Drink plenty of water to stay hydrated	83.8	15.4	0.8
Go for a swim to cool down	16.1	54.9	29.0
Wear-dark coloured clothes when going outside	1.5	30.7	67.8
Listen to daily weather forecast	61.4	35.6	3.0
Drink a few cups of coffee to stay alert	4.49	33.7	61.8
Wear a hat when going outside	56.2	32.6	11.2
Do some outdoor gardening during the day	0	23.2	76.8
Seek protection of shady areas when outdoor	82.0	16.9	1.1
Go to a shopping centre to cool down	6.0	64.0	30.0
Use an umbrella when walking outside	5.3	21.7	73.0

### 4.5. Predictors of Risk Perception to Heat Waves

Table 7 shows results of the univariate and multivariate logistic regression analyses for predictors of risk perception to the heat wave scenario. In univariate analyses, those who were married (OR = 0.29; 95% CI, 0.12–0.71), who earned a gross annual household income of ≥$60,000 (OR = 0.35; 95% CI, 0.18–0.67), without a fan (OR = 0.26; 95% CI, 0.09–0.66) were less likely to have a high risk perception to heat waves. In the multivariate model, age was a significant predictor of risk perception to heat waves (OR = 1.04; 95% CI, 1.00–1.07). In addition, those who were married (OR = 0.21; 95% CI, 0.07–0.62), those who earned a gross annual household income of ≥$60,000 (OR = 0.41; 95% CI, 0.17–0.94), and those without a fan (OR = 0.29; 95% CI, 0.11–0.79) were less likely to have a high risk perception to heat waves. However, those who were living with others (OR = 2.87; 95% CI, 1.19–6.90) were more likely to have a high risk perception to heat waves. We did not find any significant relationship between level of education or knowledge about heat waves and risk perception.

**Table 7:** Univariate and multiple logistic regression analyses for predictors of risk perception to a heat wave scenario.

Predictor variables	Category	Univariate		Multivariate	
		OR(95% CI)	p-value	OR (95% CI)	p-value
Age	NA, *continuous*	1.02(0.99–1.05)	0.071	1.04(1.00–1.07)	0.025*
Gender	Male	1(ref)		1(ref)	
	Female	1.01(0.62–1.63)	0.979	1.03(0.60–1.78)	0.897
Marital status	Not married	1(ref)		1(ref)	
	Married	0.29 (0.12–0.71)	0.007 *	0.21(0.07–0.62)	0.005 *
Educational level	≤HSD	1(ref)		1(ref)	
	>HSD	0.91(0.55–1.48)	0.699	1.13(0.63–2.04)	0.662
Employment status	Not employed	1(ref)		1(ref)	
	Employed	0.63(0.32–1.22)	0.175	1.04(0.45–2.39)	0.909
Gross annual household income	<$40,000	1(ref)		1(ref)	
	$40k– <60,000	0.69(0.32–1.48)	0.341	0.90(0.36–2.24)	0.823
	≥$60,000	0.35(0.18–0.67)	0.001 *	0.41(0.17–0.94)	0.037 *
Living arrangements	Alone	1(ref)		1(ref)	
	Others	0.94(0.49–1.79)	0.846	2.87(1.19–6.90)	0.019 *
Own a fan	Yes	1(ref)		1(ref)	
	No	0.26(0.09–0.66)	0.005 *	0.29(0.11–0.79)	0.015 *
Own an air-conditioner	Yes	1(ref)		1(ref)	
	No	0.71(0.26–1.92)	0.503	0.81(0.24–2.66)	0.733
Knowledge about heat waves	NA, *continuous*	1.01(0.56–1.80)	0.991	1.08(0.86–1.36)	0.461

* Significant at p < 0.05; ≤HSD refers to “partial or full completion of high school”; >HSD refers “additional training/education after high school”; $40k refers to $40,000 and OR refers to odds ratio; NA means “not applicable”; “ref” means “reference category”.

### 4.6. Predictors of Adaptive Behaviours during a Heat Wave

Table 8 shows the results of the logistic regression analyses for the predictors of adaptive behaviours during a heat wave. In univariate analyses, perceived benefit, “cues to action” and knowledge about heat waves were significant predictors of adaptive behaviours during a heat wave. The results suggested that those who had a high perceived benefit (OR = 2.69; 95% CI, 1.40–5.18) and high “cues to action” (OR = 2.13; 95% CI, 1.09–4.16) were more likely to have good adaptive behaviours during a heat wave. Furthermore, those who were married (OR = 2.44; 95% CI, 1.03–5.78), who had additional training or education after high school (OR = 3.03; 95% CI, 1.57–5.83) and who earned a gross annual household income of ≥$60,000(OR = 3.07; 95% CI, 1.43–6.56) were more likely to have good adaptive behaviours during a heat wave.

**Table 8:** Univariate and multiple logistic regression analyses for predictors of adaptive behaviours during a heat wave.

Predictor variables	Category	Univariate		Multivariate	
		OR (95% CI)	p-value	OR (95% CI)	*p*-value
Risk perception	Low	1(ref)		1(ref)	
	High	0.68(0.36–1.30)	0.249	0.66(0.29–1.46)	0.305
Perceived benefit	Low	1(ref)		1(ref)	
	High	2.69(1.40–5.18)	0.003 *	2.14(1.00–4.58)	0.049 *
Perceived barriers	Low	1(ref)		1 (ref)	
	High	0.49(0.23–1.09)	0.081	0.82(0.31–2.13)	0.682
Cues to action	Low	1(ref)		1 (ref)	
	High	2.13(1.09–4.16)	0.027 *	3.71(1.63–8.43)	0.002 *
Knowledge about heat waves	NA, *continuous*	1.40(1.09–1.78)	0.007 *	1.22(0.91–1.62)	0.173
Age	NA, *continuous*	1.01(0.97–1.05)	0.426		
Gender	Male	1(ref)			
	Female	0.62(0.32–1.21)	0.160		
Marital status	Never married	1(ref)		1(ref)	
	Married	2.44(1.03–5.78)	0.042 *	1.67(0.59–4.66)	0.326
Educational level	≤HSD	1(ref)		1(ref)	
	>HSD	3.03(1.57–5.83)	0.001 *	2.65(1.25–5.58)	0.010 *
Employment status	Not employed	1(ref)			
	Employed	0.75(0.29–1.88)	0.536		
Gross annual household income	<$40,000	1(ref)		1(ref)	
	$40k–<$60,000	1.78(0.73–4.34)	0.203	1.74(0.65–4.68)	0.271
	≥$60,000	3.07(1.43–6.56)	0.004 *	2.66(1.07–6.56)	0.035 *
Living arrangements	Alone	1(ref)			
	Others	0.72(0.28–1.82)	0.491		
Own a fan	Yes	1(ref)			
	No	0.67(0.25–1.77)	0.420		
Own an air-conditioner	Yes	1(ref)			
	No	0.62(0.13–2.82)	0.541		

* Significant at *p*-value < 0.05; ≤HSD refers to “partial or full completion of high school”; >HSD refers “additional training/education after high school”; $40k refers to $40,000 and OR refers to odds ratio; NA means “not applicable”; “ref” means “reference category”.

In the multivariate model, those with a high perceived benefit (OR = 2.14; 95% CI, 1.00–4.58), a high “cues to action” (OR = 3.71; 95% CI, 1.63–8.43), who had additional training or education after high school (OR = 2.65; 95% CI, 1.25–5.58) and who earned a gross annual household income of ≥$60,000(OR = 2.66; 95% CI, 1.07–6.56) were more likely to have good adaptive behaviours during a heat wave. Risk perception was not found to be a significant predictor of adaptive behaviours, a finding which did not support our hypothesis. However, the other constructs of the HBM: perceived benefit and “cues to action” were significant predictors of adaptive behaviours, which support the predictive value of some constructs of the HBM. 

## 5. Discussion

Heat wave is an environmental hazard that poses a threat to human health and the extent to which individuals perceive risks associated with heat waves may influence their decision to modify their behaviour during a heat wave. This study used an extreme heat wave scenario to assess public risk perception and identified the factors that are associated with risk perception to heat waves. In addition, the study also tested the validity of the health belief model in predicting the adoption of healthy behaviours during a heat wave. 

This study found that more than half (67.4%) of the participants had a high perceived vulnerability to the heat wave scenario. In 2009, Adelaide recorded two heat waves and during the first, daily maximum temperatures ranged from 35 °C to 45.7 °C and there were eight consecutive days with temperatures over 35 °C during the second heat wave [17]. As previously indicated, in early 2013, Australia sweltered under an unprecedented summer heat wave which set a new record temperature of 40.3 °C; the highest national daily average temperature ever recorded [16]. This is the first published study in which most participants reported feeling vulnerable to heat waves. Previous studies on perception of heat waves found contrasting results as most participants did not consider themselves vulnerable to heat waves [18,33], although the contexts in which these studies were conducted differed from the present study. A possible explanation for the present finding could be because participants felt that a heat wave characterised by 45 °C for seven consecutive days would be quite extreme. This finding could be important in public risk communication for heat waves under future climate scenarios. It has been projected that the number of days with temperatures above 40 °C in Adelaide will range from three to eight days by 2050 [49]. It is important to note that scenarios have widely been used in studies related to climate change and are extremely useful in studying the uncertainties that surrounds climate change [50].

Our study found that age, marital status, gross annual household income, living arrangements and fan ownership were significant predictors of risk perception. This finding is somewhat consistent with those of previous studies that reported an association between age and risk perception to environmental hazards [28,51]. Generally, age is an important factor for vulnerability to heat waves since those who are older are known to be at risk during a heat wave [52]. A study conducted to examine the Australian public’s perceived risks on a number of environmental hazards found that older participants had a high risk perception [30]. Nonetheless, contrasting findings between age and risk perception to environmental hazards has been reported in another study [53]. Our results suggest that those who were married were less likely to have a high risk perception to heat waves. Those who are married may believe that they have social contacts and bonds which they consider to be a protective effect against heat-related morbidity and mortality [19,24]; and this could explain why they were less likely to have a high risk perception to heat waves. Some studies have found that those who are married are less likely to suffer from heat-related illnesses and mortality [54,55].

Our study also found that those with a higher household income were less likely to have a high risk perception to heat waves. This finding is consistent with those of other studies which have found that individuals with a higher income and an overall higher socio-economic status are less likely to perceive risks as threatening [31,32]. Studies have found that those living alone are at higher risk of heat-related morbidity and mortality during a heat wave [22,56]. However, our study found that those who were living with others were more likely to perceive a high risk to heat waves. Although there is no ready explanation for this finding, one may hypothesise that those who lived with others could have expressed a high risk perception because they may also be concern about the safety and well being of their immediate family members (e.g., partner, children) and relatives [41] during such an extreme heat wave. 

With regard to adaptive behaviours, we found that two of the constructs of the HBM were significant predictors of adaptive behaviours during a heat wave. Individuals with a high perceived benefit were more likely to have good adaptive behaviours during a heat wave. This group of individuals actually recognised the benefit of taking preventive measures to stay safe during a heat wave. It was also found that “cues to action” was a significant predictor of adaptive behaviours during a heat wave. For example, a majority of the participants either strongly agreed or agreed that as a result of their personal experience of heat waves in Adelaide, they would take preventive actions to keep themselves safe during such a heat wave. This finding may suggest that previous experience from an environmental event (e.g., a heat wave) that inflicts damage may precipitate heightened risk perception and can act as a trigger for future preventive actions [57]. However, our study did not find any significant relationship between risk perception and adaptive behaviours during heat waves. A possible explanation could be because the context in which the two variables were measured was not the same. It further highlights that risk perception alone does not necessarily predict preventive behaviours [58,59] but that other factors need to be taken into consideration. Nevertheless, it is important to note that while some individuals may have a heightened risk perception to heat wave, there may be certain barriers that may hinder them from adequately adapting during a heat wave. This assertion could be supported by the finding from this study that participants who had high perceived barriers (although not a significant predictor) were less likely to have good adaptive behaviours during a heat wave.

In addition to level of education, household income was found to be a significant predictor of adaptive behaviours during a heat wave; as those with a higher income were more likely to have good adaptive behaviours. It could be inferred that individuals with a higher income are more likely to run their air-conditioners while at home during a heat wave to stay cool without the fear of electricity costs. Previous studies have reported that the high costs of running an air-conditioner may act as a barrier for certain individuals to properly adapt during a heat wave [3,18].

Overall, this study has generated some important findings about risk perception and adaptation to heat waves under a changing climate. The study is novel because it is the first to apply the HBM in assessing risk perception to heat waves within the context of a scenario; and to identify the predictors of risk perception and adaptive behaviours during heat waves using logistic regression analyses. Nonetheless, it is important to acknowledge some limitations from this study. Firstly, the present results should be interpreted with caution as they cannot be generalised to the entire Adelaide population, since the sample comprised of a sub-group of the NWAHS participants who voluntarily expressed interest to participate in the study. Secondly, the questionnaires were self-completed and therefore the analysis was based on self-reported data, for which there may have been response bias. Thirdly, although the constructs of perception (applied in the HBM) were used as explanatory variables to predict adaptive behaviours, the conditions under which the statements to assess perception of heat waves and adaptive behaviours were not the same. While the statements to assess perception were posed within the context of a scenario, those statements regarding adaptive behaviours were not under the same context. Fourthly, the study may have been limited by selection bias; the fact that some categorical demographic variables were dichotomised due to sample size constraints might have affected the results. Lastly, non-response bias might have affected the results since respondents and non-respondents differed by age.

Despite these limitations, these findings are useful for emergency service providers, health officials, local authorities and policy makers in the design and implementation of risk communication and behaviour change promotion strategies related to heat waves. This is particularly important given that climate models project more frequent heat waves in the future. The study is also useful for researchers as it serves as the basis for similar studies to be replicated in other cities and regions around the world that usually experience heat waves. Such studies would shed light on any differences in risk perception to heat waves that exist across regions. Future research should examine how participants’ threat appraisal and coping appraisal (self-efficacy) to heat waves could better predict adaptive behaviours during heat waves. More research is also needed to understand the relationship between risk perception and risk communication in the context of heat waves.

## 6. Conclusions

There is considerable uncertainty about the magnitude of future climate change impacts. However, scientific projections maintain that heat waves will likely increase in frequency, intensity and duration [1,2]. This research has enabled us to estimate risk perception of a heat wave scenario through perceived vulnerability and perceived severity. By examining risk perception in the context of a heat wave scenario, this paper increases our understanding on the extent to which individuals evaluate the risk associated with an extreme heat wave. Although the context applied in this research was hypothetical, it may be likely that such a scenario could be experienced in future. After all, climate models project temperature rises across most Australian cities with an increase in duration of heat waves [49]. Our finding that age, marital status, gross annual household income, fan ownership and living arrangements were significant predictors of risk perception in the context of the heat wave scenario supports the existing scholarship that there are certain socio-demographic factors that influence risk perception to environmental hazards [25,26,27]. In our study, we did not find any significant association between risk perception and adaptive behaviours during heat waves. Nevertheless, this research has provided emergency management agencies with relevant information about the factors that must be considered when they develop and disseminate risk communication messages related to heat waves both for current and future climate scenarios.

Our research is particularly useful in the sense that it applied a behaviour change theory to identify the factors that could predict the adoption of healthy behaviours during a heat wave. The study has demonstrated that factors such as knowledge about heat waves, marital status, level of education, gross annual household income are significant predictors of adaptive behaviours during a heat wave. In addition, the findings that perceived benefit and “cues to action” were significant predictors of adaptive behaviours further suggest the usefulness of the HBM in behaviour change programs for heat waves. As a result, health promotion specialists must take into account these factors and predictor variables during the design and implementation of concise and targeted health messages intended to motivate behaviour change during heat waves. Moreover, it is important that those behaviours that increase an individual’s risk to heat-related morbidity and mortality are identified such that they are effectively targeted. The perceived barriers to appropriate adaptive behaviours should be targeted by providing information to the public on the available options, providing incentives, offering reassurances and dispelling any misconceptions [38]. This will greatly determine the success of any behaviour change program for heat waves. 
